# Complete Genome Sequence of a Human Coxsackievirus B3 from a Child with Myocarditis in Beijing, China

**DOI:** 10.1128/genomeA.00163-12

**Published:** 2013-02-07

**Authors:** Jiang Du, Zhiqiang Wu, Ying Xue, Ting Zhang, Fan Yang, Qi Jin

**Affiliations:** MOH Key Laboratory of Systems Biology of Pathogens, Institute of Pathogen Biology, Chinese Academy of Medical Sciences & Peking Union Medical College, Beijing, People’s Republic of China

## Abstract

A human coxsackievirus B3 (CVB3), designated strain Beijing0811, was isolated from a child diagnosed with hospital-acquired infectious acute myocarditis in Beijing, China, and propagated in human rhabdomyosarcoma cells. The complete genome sequence of this virus was 7,402 nucleotides, excluding the 3′ poly(A) tail, which encoded a large polyprotein with 2,185 amino acids. This report will help us to analyze the evolutionary and epidemic characteristics of CVB3.

## GENOME ANNOUNCEMENT

Human coxsackievirus B3 serogroup (CVB3) is a member of the genus *Enterovirus*, which is in the family *Picornaviridae*. CVB3 is one of the most common human pathogens associated with clinical acute and chronic viral myocarditis; some acute infections are severe and lethal (1, 2, 3).

From the pharyngeal swab of a child in the city of Beijing, China, with a clinical diagnosis of hospital-acquired infectious acute myocarditis, the CVB3 strain Beijing0811 was confirmed by using reverse transcription and seminested PCR as described in a previous study (4, 5). This CVB3 strain produced typical cytopathic effects (CPE) in human rhabdomyosarcoma cells (RD cells). After propagation and plaque purification in cell culture, overlap fragments of this virus were obtained by using degenerate primers based on genome sequences of existing CVB3 strains available in GenBank, and the complete genome sequence of this virus was established by assembling overlap fragments using the SeqMan program available within the Lasergene 7 package (DNASTAR).

The complete genome of the CVB3 strain Beijing0811 (7,402 nucleotides) contained a single open reading frame (ORF) that encoded a large polyprotein (2,185 amino acids), flanked by a 5′-untranslated region (UTR) and a 3′-UTR. An alignment analysis of this virus and other known enteroviruses showed the characteristic gene order of 5′-P1 (851 amino acids [aa], VP4, VP2, VP3, and VP1), -P2 (578 aa, 2A, 2B, and 2C), -P3 (756 aa, 3A, 3B, 3C, and 3D)-3′. It also showed the theorized cleavage sites in the polyprotein. The P1, P2, and P3 polypeptides were hypothetically cleaved at VP4/VP2 (N/S), VP2/VP3 (Q/G), VP3/VP1 (Q/G), 2A/2B (Q/G), 2B/2C (Q/N), 3A/3B (Q/G), 3B/3C (Q/G), and 3C/3D (G/E).

Phylogenetic and pairwise alignment analyses based on P1, P2, and P3 regions were conducted separately by using MEGA5.0 (6). The CVB strain Beijing0811 was closely related to another CVB3 Chinese isolate, strain Fuyang19 (GenBank accession no. FJ000001, more than 98% nucleotide identities in P1, P2, and P3). Phylogenetic analysis based on P1 showed that CVB3 strain Beijing0811 was clustered with its corresponding CVB3 prototype strains, but phylogenetic analysis based on P3 showed that CVB3 strain Beijing0811 was clustered with CVB5 strains such as CVB5/CC10/17 (GenBank accession no. JN695051), CVB5/CC10/16 (GenBank accession no. JN695050), and 20CSF (GenBank accession no. JX017380). SimPlot and bootscanning analyses of this strain and other enteroviruses confirmed that recombination occurred between CVB3 and CVB5.

### Nucleotide sequence accession number.

The genome sequence of CVB3 strain Beijing0811 was deposited in GenBank (GenBank accession no. GQ141875).
